# Delivering Modern Global Health Learning Requires New Obligations and Approaches

**DOI:** 10.5334/aogh.3261

**Published:** 2021-07-12

**Authors:** Scott J. N. McNabb, Mabel Magowe, Nadine Shaw, Amanda M. Berrian, Michael Wilkes, Affan Shaikh, Onesmus Gachuno, Lucy A. Perrone, Brittany L. Murray, Eva Berman, Bhakti Hansoti

**Affiliations:** 1 Rollins School of Public Health, Emory University, Atlanta, USA; 2 School of Nursing, University of Botswana, Gaborone, Botswana; 3 Evans School of Public Policy and Governance, University of Washington, Seattle, USA; 4 International Program in Public Health Leadership; 5 College of Veterinary Medicine, The Ohio State University, Columbus, USA; 6 Global One Health initiative; 7 Global Health, Office of the Dean, School of Medicine University of California, Davis, Davis, USA; 8 School of Management, Yale University, New Haven, USA; 9 Department of Obstetrics and Gynecology, University of Nairobi, Nairobi, Kenya; 10 Afya Bora Consortium; 11 Departments of Global Health and Laboratory Medicine, University of Washington, Seattle, USA; 12 International Training & Education Center for Health (I-TECH); 13 Emory University School of Medicine, Atlanta, USA; 14 Children’s Healthcare of Atlanta, Atlanta, USA; 15 Department of Global Health, University of Washington, Seattle, USA; 16 Johns Hopkins School of Medicine, Baltimore, USA; 17 STAR Program

## Abstract

**Introduction:**

The COVID-19 pandemic has forced a new look (or modernization) for both the obligations and approaches to achieve best-practices in global health learning. These best-practices have moved beyond traditional, face-to-face (F2F), classroom-based didactics to the use of innovative online, asynchronous and synchronous instructional design and the information and communication technology (ICT) tools to support it. But moving to this higher level of online in-service and pre-service training, key obligations (e.g., stopping neocolonialization, cultural humility, reversing brain drain, gender equity) must guide the modernization of instructional design and the supporting ICT. To positively impact global health training, educators must meet the needs of learners *where they are*.

**Purpose:**

We describe a set of multi-communication methods, e-Learning principles, strategies, and ICT approaches for educators to pivot content delivery from traditional, F2F classroom didactics into the modern era. These best-practices in both the obligations and approaches utilize thoughtful, modern strategies of instructional design and ICT.

**Approach:**

We harnessed our collective experiences in global health training to present thoughtful insights on the guiding principles, strategies, and ICT environment central to develop learning curricula that meet trainee needs and how they can be actualized. Specifically, we describe five strategies: 1. Individualized learning; 2. Provide experiential learning; 3. Mentor … Mentor … Mentor; 4. Reinforce learning through assessment; and 5. Information and communication technology and tools to support learning.

**Discussion:**

We offer a vision, set of guiding principles, and five strategies for successful curricula delivery in the modern era so that global health training can be made available to a wider audience more efficiently and effectively.

## Introduction

During the COVID-19 pandemic, we see distinct gaps in the public health fabric (or architecture) that must be addressed [1]. To address them, workforce development is particularly crucial to build healthcare and public health capacity. Training needs for global health professionals are widespread and immediate. Successful delivery of learning curricula must overcome inequities (e.g., access to information and communication technology (ICT), learning resources, transportation, competing priorities, gender prejudice, insufficient mentorship, and limited numbers of champions for training). In 2021, barriers to training are compounded by embargos on travel and in-person, face-to-face (F2F) activities.

There are key guiding principles supporting best-practices that move in-service, global health training into the modern, post-COVID-19 era (***Table 1***). Unlike pre-service education—focused on undergraduate or graduate education—in-service training of global health professionals must be nuanced and nimbler to respond to the wide breadth and depth of experience and expertise. For example, the USAID-sponsored Sustaining Technical and Analytic Resources (STAR) supports more than 50 fellows in 38 countries. The roles of these individuals vary from senior technical advisors managing health programs in numerous countries to communication heads that focus on developing strategies with a single implementing partner organization.

**Table 1:** Principles Guiding Learning Strategies for Global Health Training.


Cultural humility and servant leadership

Gender equity

Ethical collaboration and knowledge sharing

Overcoming academic, administrative, and topical silos

Reversing brain drain

Transparent learning networks

Respecting intellectual property

Drive toward One Health

Creating multi-communication, digital environments that support the real world

Building informatics-savvy organizations to overcome learning barriers

Achieving an educational environment where participants want to participate


The challenge becomes how to meet learners *where they are* and support them so that their training becomes not a burden but an advantage to their work-life and work-based priorities [2]. This is even more challenging when training moves beyond technical knowledge or skills-focused training to professional training geared toward building global health leaders, which relies on the development of communication skills, cross-cultural practice, development practice, and so forth. Global health is as varied, unique, and complex as the professionals within it, and learning programs need to be dynamic to actively encourage the building of learning networks to promote knowledge sharing and connectivity [3].

Lastly, there is a need to make training open and globally accessible. The new Africa CDC began training the continent’s workforce on a large scale using a variety of multi-communication, e-Learning platforms. After conducting an extensive literature review of e-Learning to improve workforce capacity, Africa CDC designed, developed, and deployed the Institute for Workforce Development (IWD) to provide high-level, in-service training for African health workers in field epidemiology, public health informatics, and laboratory science. Beyond a resource library of four courses, the Africa CDC IWD has also deployed a Clinical Community of Practice (CCoP) and connected the clinical community to weekly webinars, office hours, and a Telegram™ group chat to address the medical challenges of the COVID-19 pandemic.

Technology-based solutions have been central to responding to the evolving needs of global heath training [4]. Using various platforms (e.g., Facebook™, Instagram™, Twitter™, LinkedIn™, and Telegram™), the Africa CDC IWD presented scientific information and supported knowledge sharing. A variety of platforms were leveraged to support knowledge transfer, including those for media hosting (e.g., Canvas™); management tools (e.g., Asana™ and Slack™); business conferencing (e.g., Zoom™); and mobile apps (e.g., Facebook™, Telegram™, and WhatsApp™).

We harnessed our collective experience in global health education and leadership training to present here thoughtful insights on the guiding principles, strategies, and ICT environments central to strengthening learning curricula that meet the needs of global health trainees, in particular senior health professionals focused on in-service and leadership training. Specifically, we describe five strategies: 1. Individualized learning; 2. Provide experiential learning; 3. Mentor … Mentor … Mentor; 4. Reinforce learning through assessment; and 5. Information and communication technology and tools to support learning.

## Strategy 1. Individualized learning

The global health workforce has pressing and continually evolving training needs, and in-service trainees often report a wide breadth of experience with knowledge gaps [5]. Meaningful engagement must meet learners *where they are* [2]. The STAR program developed a learning strategy that includes developing an individualized learning plan (ILP) for each participant, creating a highly tailored learning experience based on factors such as career goals, identifying individual learning preferences, and respecting the needs of the host institution where participants are based [6].

Supporting the learner as an individual requires consideration of training content, as well as implementation strategy. For adult learning, there is a need to incorporate individualism. Adult learners often have the self-concept of being responsible for their own decisions, in part due to the self-determination theory for motivation [7,8]. A learner-centered curriculum (opposed to a program-centered) will result in buy-in and engagement.

Another strategy is to use tools that encourage learners to explore a topic independently, moving from teacher-centered instruction and maximizing autonomy. Used effectively, this allows learners not only to achieve the learning objectives, but also to develop skills critical for lifelong learning. Other learner-centered approaches include

- applied learning approaches.
  Adult learners gravitate toward learning that provides benefit. Educators should strive to provide learners the opportunity to absorb and apply information, rather than memorize it. An additional strategy is to weave in real-life applications and, where possible, encourage trainees to bring their work to the classroom.
- orienting learning to the immediate future.
  Emphasis should be on subject matter that assists in solving problems encountered regularly. Benefits to learning must be addressed; educators should understand the current and future professional roles of their learners and integrate related competencies into learning objectives, while identifying how the learning can support professional growth or advancement.
- motivation to learn.
  Intrinsic motivation is key for adult learners; strive to provide a valid justification behind every educational activity. Incorporating a wide range of instructional design strategies, including active, hands-on learning experiences, can appeal to varied experiences and backgrounds and motivate authentic learning, but the range of approaches should be justified and not burdensome for the sake of being different or new.
- promoting active learning.
  Educators should seek to create engaging, experiential learning management architecture and content. John Dewey, an educational philosopher, supported this vision long ago: “[Teachers] give the pupils something to do, not something to learn; and the doing is of such a nature as to demand thinking, or the intentional noting of connections; learning naturally results [9]”. Problem-based learning or pathfinder algorithms are two strategies that provide learning through critical thinking and analysis.
- open-access learning.
  Knowledge sharing beyond the classroom to one’s institution, colleagues, and mentees is key not only to improving engagement, but also to reinforcing learning content. Beyond the sharing of learning resources, the sharing of real-world data, indicator-based reporting, pathogen isolates, and clinical specimens allows learners to engage with the subject matter beyond the classroom.

As educators, we have a mandate to develop innovative strategies to deliver learning in meaningful ways. To meet learners *where they are*, global health programs and institutions have employed strategies to stimulate meaningful engagement. The Afya Bora fellowship reinforces didactic modules to include real-world, case-based learning, which provides the opportunity to work through problems with diverse groups in terms of background, gender, and nationality. Further, problem solving with a diverse group of stakeholders is central to effective public health and allows the learning to be immediately transferable to the immediate work environment [10].

## Strategy 2. Provide experiential learning

Massive Open Online Courses (MOOCs), while widely available, have been limited in impact due to the one-dimensional nature of content delivery. Blended e-Learning programs that utilize a combination of in-person and online training provide learners a richer experience stemming from close interaction with peers and faculty. Effective competency-based, in-service education programs have also been highly useful in developing the healthcare workforce. The best strategy incorporates an applied assignment that runs the duration of the e-Learning program, enabling participants to apply theory learned from the global classroom directly to their work. Individualized projects allow learners to address relevant challenges to their jobs and work to address a topic of significance for their own careers; these can include proposal development or implementation evaluations.

Development of a customized project builds critical thinking skills and teaches the practices of continuous quality improvement when projects are designed for healthcare settings, such as clinical laboratories. Incorporating an assignment like this results in stronger engagement by participants in an e-Learning program. Further, it builds communities of practice through cycles of peer and mentor review of periodic assignments, which can be built into the program and punctuate breaks in online coursework.

For example, in the I-TECH certificate program in laboratory leadership and management (CPLLM), a nine-month blended, e-Learning program includes five online courses and an applied capstone project [11]. Programs like these that incorporate an applied assignment have lower rates of attrition (e.g., 86% graduation rate in the CPLLM). Blended e-Learning and competency-based programs also have facility-level impact. Results from a CPLLM cohort in Zambia showed that through their capstone projects, all participants improved their laboratory’s compliance with international standards for quality, and five facilities achieved international accreditation.

## Strategy 3. Mentor… Mentor… Mentor

Mentorship provides guidance, encouragement, and support from experienced professionals to the less experienced and can contribute to the personalization of educational experiences. Mentorship is reciprocal and allows the mentor and mentee to collaborate and work on the mentee’s goals [12]. Within educational programs, mentorship can also help place learning in context by using shared professional experiences to create a scaffolding for the mentee to understand the importance of new information.

This model aligns with adult learning theories and can help motivate mentees to pursue and complete professional development activities [7,13,14]. Mentors can also help mentees understand and explore the culture and unspoken rules of a profession; this assists mentees in career choices that lead to personal fulfillment, professional advancement, and success. While mentees may benefit from the recruitment of several mentors to address different areas of their professional life (e.g., clinical, research, work-life balance), most mentoring relationships still build on the traditional elbow-to-elbow mentoring model [12,15]. The types of commonly encountered mentorship types include the following:

- expert (1:1 mentorship, traditionally face-to-face, but can be adapted online).
- supervisory (mentorship provided by a direct supervisor).
- peer (peers sharing knowledge of specific experiences).
- group (single mentor working with a group of mentees).
- hybrid (combination of mentorship methods).

The difficulties global health educational initiatives face in incorporating mentorship programs include the lack of availability of on-site mentors (both in the number of senior professionals available and the competing priorities for their time) and differing cultures and perceptions of professional mentorship [16,17,18]. These barriers can be overcome, at least in part, by pragmatic program design, clear delineation of roles, and the incorporation of ICT. In all mentorship relationships, clearly defining the roles and responsibilities of both mentors and mentees is key to helping mentorship relationships flourish, especially when moving beyond traditional one-on-one relationships [19].

Online mentoring strategies can be adopted to overcome the limitations of local availability of mentors but building connections is challenging. There are well-established tools developed to support initial connections and investments and to identify shared interests. Hybrid mentorship models that combine multiple approaches can be leveraged to overcome the limitations of any single one, for example facilitated group mentorship within the immediate work environment to promote knowledge sharing, reinforced by remote mentoring that is focused on transferring expertise not available locally, as was adopted by the STAR project [6].

## Strategy 4. Reinforce learning through assessment

Assessment means measuring learner performance. Education is complex with learning delivered in a variety of formats (e.g., didactic, e-Learning, small group, at the bedside, simulation centers). Further, learning occurs in a variety of locations (e.g., field sites, hospitals, clinics, classrooms, laboratories, simulation centers). Traditional assessments use tools such as pre- and post-tests to quantify change in knowledge and to track achievement of competencies. In global health, knowledge is not the only domain that drives competency. Other valid measures of assessment that capture acquisition of skills, attitudes, and behavior should be incorporated into training programs.

The current assessment toolbox is large and contains a variety of practical examinations (e.g., Objective Structured Clinical Exams [OSCE], Objective Structured Video Exercise [OSVE], simulation exercises, evaluation and assessment of standardized patients, computer-based assessments, pathfinder assessments). A change in how, when, where, and with whom assessment occurs requires a paradigm shift in thinking. Clear, growth-oriented assessment is rarely reduced to a precise numerical grade but is more likely to involve assessments with specific narrative feedback, reflection, and the development of a clear action plan to guide future development.

A single approach to assessment, such as multiple-choice exams, are limited in their ability to capture anything beyond change in knowledge. Futher, studies show that knowledge-based exams correlate poorly with future skills and behaviors [20]. High-quality assessments contextualize how learned material can be applied to its eventual use. One such strategy is to obtain a 360-degree assessment, which generally incorporates self-assessment as well as feedback from supervisors, peers, and mentees into the assessment strategy.

Many scholars, including Miller (knows, knows how, shows how, does); Bloom (knowledge/cognitive, skills/psychomotor domain, attitude/affective domain); and Dreyfus (novice, advanced beginner, competent, proficient, expert, master) created schemes to describe the stages of learning that, in turn, allow for appropriate individual assessment [21]. Each level of proficiency has a narrow definition relative to the material that allows for development of an educationally sound learning plan, appropriate assessment, and the creation of individual action plans to guide future progress.

Following assessments, learners are taught reflection, self-assessment with action planning, and peer assessment. Because practicing health professionals must constantly update their knowledge and evaluate their competency, they need to determine their skill level and what and how they will acquire new skills. These lifelong learning proficiencies require being well trained in self-assessment—its uses and limitations. In addition, schools devote a great deal of time to faculty development, promoting skills to provide learners effective goal-directed feedback and the development of action plans.

The journey towards lifelong learning begins with an inward reflection of one’s thoughts, feelings, and behaviors. Reflection leads to accepting responsibility for your own thinking, treatment planning, actions, and outcomes, plus understanding the motivations for self-learning. The use of the Self-Reflection and Insight Scale (SRIS) has proven effective for self-directed change, improved self-regulation, and improved patient outcomes and has strengthened the assessment approach to translate into behavior change [22,23,24].

## Strategy 5. Information and communication technology and tools to support learning

The e-Learning experience—defined as learning conducted online—is not equal for all. A learner’s experience (i.e., ability to engage content *where they are*) is tied to internet access and tools (e.g., learning management system, media suite), devices (e.g., mobile phone, laptop, desktop), and dedicated space and time away from other obligations and distractions. The quality of the learning access and tools (software and hardware) affects both access and facility of use.

Synchronous and asynchronous, multi-communication, e-Learning supports the vision of *where they are*, meaning where learning is sought and gained to the meet the immediate needs of the participant; it also is at the convenience of the participant. Many learners may have family obligations or competing work/life priorities that would prevent them from engaging in traditional classroom didactics. While some learners are simply more engaged by a F2F experience, the disadvantage of an asynchronous learning experience is overcome by the convenience of accessibility. Further, the multi-communication methods availed by online and e-Learning platforms open doors for interactivity that facilitates engaged behavior (e.g., using just-in-time analytics and polls to gauge level of understanding).

No matter the platform or device, the content of e-Learning modules must conform to best practices in instructional design. Trainers must use the best modes of delivery (e.g., video, text, images, graphs, charts) that allow learners to engage with information suited to how they best learn. Most important, e-Learning makes it possible to collaborate and communicate with one another in different spaces, at different times, and it provides a level of flexibility unmatched in the F2F classroom.

## Conclusion

We present modern guiding principles that support the delivery of curricula for global health learners. Accounting for the depth and breadth of experiences that participants have, we advocate for delivery strategies that allow learning to be targeted and customizable for the individual. Further, given recent travel restrictions imposed by the COVID-19 pandemic and the desire to improve worldwide access to curricula, we discuss the specific advantages and challenges to e-Learning. While e-Learning platforms provide opportunities for wider reach and increased flexibility for access, they also demand effort and consideration to deliver high-yield content. Instructional design strategies must facilitate application of e-Learning, as well as connectivity to peers and experts.

Programs with a heavy reliance on e-Learning modalities will be strengthened by incorporation of mentorship programs and experiential opportunities. Bolstering curricula with holistic assessment approaches, including mentorship, provides greatest yields by introducing concepts for self-reflection and feedback. The approaches we emphasize stress quality of learning, learner engagement and support, and access to meet the learner *where they are*.
